# Delivery of stromal cell-derived factor 1α for *in situ* tissue regeneration

**DOI:** 10.1186/s13036-017-0058-3

**Published:** 2017-06-29

**Authors:** Wen Zhao, Kaixiang Jin, Jiaojiao Li, Xuefeng Qiu, Song Li

**Affiliations:** 1 Key Laboratory for Space Biosciences and Biotechnology, School of Life Sciences, Northwestern Polytechnical University, Xi’an, Shaanxi 710072 China; 2 Department of Bioengineering and Department of Medicine, University of California, Los Angeles, CA 90095 USA

**Keywords:** *In-situ* tissue engineering, Stromal cell-derived factor, Cell-instructive biomaterials, Bonding interaction, Chemokine

## Abstract

*In situ* tissue regeneration approach aims to exploit the body’s own biological resources and reparative capability and recruit host cells by utilizing cell-instructive biomaterials. In order to immobilize and release bioactive factors in biomaterials, it is important to engineer the load effectiveness, release kinetics and cell recruiting capabilities of bioactive molecules by using suitable bonding strategies. Stromal cell-derived factor 1α (SDF-1α) is one of the most potent chemokines for stem cell recruitment, and SDF-1α-loaded scaffolds have been used for the regeneration of many types of tissues. This review summarizes the strategies to incorporate SDF-1α into scaffolds, including direct loading or adsorption, polyion complexes, specific heparin-mediated interaction and particulate system, which may be applied to the immobilization of other chemokines or growth factors. In addition, we discuss the application of these strategies in the regeneration of tissues such as blood vessel, myocardium, cartilage and bone.

## Background

Tissue engineering combines the knowledge and technologies in engineering, biology and medicine to promote the regeneration of tissues and the restoration of tissue and organ function. In the past two decades, the approaches of tissue engineering have evolved to facilitate the translation of research findings and technologies into clinical applications [1–3]. A classical approach of tissue engineering is to fabricate bioengineered tissues or organs by culturing allogeneic or autologous cells on the scaffold in vitro, followed by the implantation of the cellular constructs. However, this strategy presents several notable disadvantages: cell culture is costly and time-consuming; there may be a phenotypic change of the cells during cell expansion, cellular construct has limited shelf life and is vulnerable to contamination; and only a fraction of seed cells actually contribute to tissue formation. Recent progress in tissue engineering and regenerative medicine has resulted in the adoption of the concept of the utilization of cell-instructive biomaterials with bioactive molecules for *in situ* tissue engineering [4, 5].

Rather than implanting cells or tissue grown in vitro, *in situ* tissue engineering involves the implantation of bioactive scaffold material decorated with, or eluting, bioactive factors into the tissue defect in order to engage the natural regeneration capacity of the host by recruiting stem cells or progenitor cells. In some types of tissues, the number of adult stem cells surrounding an implanted scaffold may be too low to have a significant impact on the acceleration of tissue regeneration [4]. Recent studies have proven that stem cells from the blood circulation can play a significant role in vascularization, hematopoiesis and mesenchymal tissue regeneration [6, 7]. Therefore, it is also valuable to mobilize progenitors from the peripheral blood system.

Stromal cell-derived factor-1α (SDF-1α) is a member of the CXC chemokine family of pro-inflammatory mediators and a potent chemoattractant for a variety of cells, especially CXC chemokine receptor type 4 (CXCR4) positive progenitors [8, 9]. Upon injury, cells from the injured tissue express and release a high level of SDF-1α, which causes a concentration gradient of SDF-1α from injured tissue to the surrounding microenvironment. CD34+ progenitor cells from the peripheral blood circulation can be recruited via chemotactic attraction toward this gradient [10, 11]. Some investigators have also reported on the role of SDF-1α in the mobilization and recruitment of bone marrow-derived hematopoietic stem cells (HSCs) and mesenchymal stem cells (MSCs), which contribute to the regeneration of blood vessels, bone, cartilage, skeletal muscle [12–15]. Some researchers have noted that the existence of SDF-1α around an *in-situ* tissue regeneration scaffold induces cell migration to the scaffold [15, 16]. Therefore, incorporation of SDF-1α into a suitable tissue engineering scaffold is an effective method of recruiting host circulating stem cells to the target tissue.

Both loading capacity and the release property of SDF-1α are critical for tissue regeneration. All of the controlled release characteristics are dependent on how SDF-1α is incorporated into the scaffolds. Hence, this review summarizes various bonding strategies of SDF-1α in biomaterials. Additionally, the typical applications of SDF-1α-loaded scaffolds in the regeneration of blood vessels, myocardium, cartilage and bone are discussed.

## Bonding strategies

In order to achieve *in situ* tissue regeneration, the release kinetics, loading efficiency and quantity of SDF-1α-loaded scaffolds can be engineered through different bonding strategies. Generally speaking, bonding between scaffolds and SDF-1α can be classified as physical and chemical immobilization. Importantly, the premature degradation of SDF-1α should also be prevented [17, 18]. Table 1 provides a summary of SDF-1α bonding strategies that have been developed.

**Table 1:** Bonding strategies of SDF-1α-loaded scaffolds

	Bonding strategy	Features	Applications (SM; LC; LE)	References
	Direct loading or adsorption	Ease of operation; burst release; short release duration; poor loading efficiency	hydroxyethyl methacrylate (HEMA) /hyaluronic acid (HA) hydrogels; SDF-1α: 4 μg/ml	[31]
			PCL and type B-gelatin; SDF-1α:2.5 ~ 10 μg/ml	[19]
	Immobilization through the formation of ionic complexes	Extensive applicability; efficient dsorption; free of linker molecules; less dependent on surface properties; adjustable release rate; requires cytotoxic surfactants	PGS (PEDA/heparin coacervate); SDF-1α:8 μg/ml Efficiency:94.3%	[51]
			PPCN; SDF-1α:0.5 μg /ml Efficiency:102.8%	[49]
	Immobilization through specific heparin-mediated interaction	Anti-thrombogenicity; efficient adsorption; prevent enzymolysis; sustained release; complex operation	19%PLLA 5%PCL (w/v); SDF-1α:0.5 μg /ml	[47]
			StarPEG-heparin hydrogel; SDF-1α:2.5 ~ 15 μg/ml; Efficiency:99.6%	[57]
			Co-Cr plates; SDF-1α:0.25 ~ 2 μg/ml	[61]
	Particulate systems	Sustained release; long release duration; multiple proteins load; complex operation	Dex-GMA/gelatin microcapsules (PNIPAAm thermo gates); SDF-1α:0.1 μg /ml Efficiency:97.5%	[20]
			PLGA nanoparticles	[27]

*Abbreviations*: *SM* scaffold materials, *LC* loading concentration, *LE* loading efficiency

Compared with chemical bonding, physical adsorption is weaker, and has a burst release and short release duration. Ji et al. suggests that this initial burst release of SDF-1α is responsible for a more effective recruitment of stem/progenitor cells and so conducive of superior clinical outcome [19]. However, Chen et al. holds the opposite point of view [20]. They emphasize that the rapid elution of SDF-1α may lead to some adverse effects. For example, SDF-1α can be cleaved by various enzymes including dipeptidylpeptidase-4 (DPP-4), metalloproteinases (MMPs) , neutrophil elastase and cathepsin G, leading to the generation of neurotoxic products which known to be involved in some forms of dementia [21–25].

Chemical immobilization of bioactive factors onto the surface of scaffolds is generally superior to physical immobilization in tissue engineering applications to prevent them being washed out when the scaffold is in contact with fluid over an extended period. Furthermore, the loading efficiency of chemically immobilized bioactive factors is generally higher, which avoids wasting bioactive factors during fabrication. However, denaturation and the loss of bioactivity might happen during chemical immobilization due to exposure of the loaded bioactive factors to organic-aqueous interfaces [26, 27]. In addition, the loading procedure is more complicated than for physical adsorption.

As the synergistic effects between SDF-1α and multiple chemokines have been observed [28], selecting appropriate bonding strategy for each of the bioactive factors is also challenging.

Here we review typical bonding strategies SDF-1α immobilization. We also introduce some technologies that can be used for the loading of SDF-1α.

### Direct loading or adsorption

Direct loading or adsorption of bioactive factors onto biomaterials is widely used. In this case, chemokines such as SDF-1α are incorporated during the fabrication process of the scaffolds, especially hydrogels, because the reaction process is relatively mild allowing chemokine bioactivity to be retained [29–32]. Alternatively, physical adsorption can be done by immersing porous scaffolds in a solution of SDF-1α or injecting SDF-1α into the scaffold [33–35]. The incorporated SDF-1α can be released upon the desorption from the scaffold or the degradation of the scaffold. The release kinetics of this type of scaffold shows a burst release during the first few hours and subsequently stable release over the following few days [19]. However, the loading efficiency of this kind of scaffold is usually poor.

Some researchers have attempted to improve the adsorption efficiency of protein-loaded scaffolds by some specific methods that could be employed to load SDF-1α into a scaffold. For instance, Koh and his colleagues [36] used inductively-coupled radio-frequency glow discharge plasma, normally used to clean biomaterials, to improve the poor loading efficiency of the physical adsorption process. The plasma could trigger a reaction with polymer scaffolds and break the chemical bonds on the surface. Thus, the surface reactivity of scaffolds was increased, making it easy for bioactive factors to be absorbed upon the immersion of the scaffolds in a solution of chemokine.

Direct loading or adsorption processes are relatively simple and time-saving. However, the burst release kinetics, short release duration and poor loading efficiency limit its application.

### Immobilization through the formation of polyion complexes

Polyion complexes are formed by electrostatic interactions between charged polyelectrolytes and their oppositely charged partners [37, 38]. The interactions are relative stable because it would be statistically impossible for all the ionic interactions on the molecules to dissociate concurrently [39]. This approach does not require additional modification of delivery matrices or linker molecules for covalent crosslinking before the incorporation of bioactive factors. Generally speaking, polyion complexes can be used for the controlled release of multiple charged therapeutic agents such as polysaccharides, proteins, polynucleotides and oligo through their coupling to fibers [40, 41] or microcapsules [42–46]. A typical positively-charged polymer material is chitosan, while commonly used negatively-charged polymer materials include sodium carboxymethyl cellulose, sodium alginate, hyaluronic acid and polyacrylates.

Liao et al. [40] introduced the interfacial polyelectrolyte complexation technology to produce drug-loaded chitosan–alginate fibers. Bioactive agents are dispersed into either the alginate or chitosan solution prior to fiber formation. By varying the ratio of the components in the anionic or cationic polyelectrolyte solution, the release behavior of the protein or growth factor can be significantly altered. In some studies, scaffold materials were chosen with an isoelectric point (IEP) that achieved a better coating rate and loading capacity of SDF-1α [35, 47–49]. When the pH of the medium is greater than the IEP of the scaffold component, the material easily absorbs cations, and conversely, when the pH of the medium is less than the IEP, the material tends to absorb anions [47]. This theoretically allows electrostatic interactions to adjust between a charged bioactive factors and an oppositely charged molecule by changing the pH of the medium. In particular, SDF-1α has a net charge of +8 at pH 7.4 (IEP of SDF-1α: 9.9) [50], so it is more efficient to load into a negatively charged scaffold. In short, the release kinetics and loading efficiency of coupled bioactive factors can easily be modulated by modifying the ionic strength, charge density, pH and the interacting scaffold.

Lee et al. [51] used a strong polycation to neutralize excess negative charges on heparin molecules to drive spontaneous coacervation. Since coacervation is a phase separation process, the coating method is less dependent on the surface properties of the scaffold [52, 53]. Thus, SDF-1α coacervate can easily be incorporated and uniformly dispersed on the surface of poly(glycerol sebacate) (PGS) scaffolds in aqueous solution without any exogenous chemicals. Furthermore, SDF-1α-loaded coacervate did not block existing pores and created a natural SDF-1α gradient from surface to the deeper layer of the porous scaffold, allowing stem/progenitor cell homing.

Immobilization of SDF-1α through polyion complexes is free of linker molecules, is less dependent on the surface properties of scaffold, and allows easy adjustment of release rate. However, the process requires polarity matched bioactive factors and polymer materials which may limit its application.

### Immobilization through specific heparin-mediated interaction

Heparin is a highly sulfated polysaccharide which is commonly used as an anticoagulant. Through specific heparin-mediated interactions with chemokines [54], chemokines can be protected from premature degradation, playing a crucial role in maintenance of physiological chemokine function. In particular, SDF-1α binds to heparin sulfate through a typical consensus sequence for heparin recognition. Lys-1, Lys-24, Lys-27 and Arg-41 on the surface of SDF-1α are essential for its interaction with heparin [54–56].

Commonly, heparin is crosslinked with the components of the hydrogel, and SDF-1α in aqueous solutions interacts with the modified hydrogel through a specific heparin-mediated interaction [16, 57–60]. Alternatively, heparin can also be covalently linked to polymer scaffolds through linker molecules. SDF-1α is then immobilized to the conjugated heparin through its heparin-binding domain [61]. For example, Yu et al. [47] employed NH_2_-PEG-NH_2_ as a linker molecule attached to the carboxylic acid groups of microfibers, and then covalently attached heparin to the free amines of the NH_2_-PEG-NH_2_ molecules using carbodiimide chemistry [62]. Finally, SDF-1α was bound to heparin via the specific interaction between them. This approach ensures that the scaffold can recruit target cells in addition to the anti-coagulation property [54]. This study demonstrated that SDF-1α immobilization on the scaffolds was stable with a sustained release of SDF-1α over one week in vitro. In addtion, SDF-1α loading efficiency is three times higher than the direct adsorbing process [47].

In order to mimic native extracellular matrices that provide mechanical support and chemical signals, Tsurkan et al. [63] introduced a class of biodegradable hydrogel that tunes its mechanical properties by the modulation of the degree of crosslinking and degradation by a specific enzyme. Specifically, all four arms of a hydroxyl-terminated star-polyethylene glycol (sPEG) were modified with acrylate groups (forming sPEG-Acl). These acrylate groups were then coupled with an MMP-cleavable peptide sequence. In the last procedure, the N-terminal amino groups of the sPEG-peptide were linked to carboxylic groups of heparin molecules to create a three dimensional network. Bioactive factors, such as SDF-1α, could be loaded to the network through the specific heparin-mediated interaction. Furthermore, the degradation rate of the hydrogel could be further modulated by using peptides with different enzymatic sensitivity, hence expanding the application area of the SDF-1α-heparin containing scaffold.

Compared with direct loading or adsorption, loading efficiency of SDF-1α is improved dramatically due to electrostatic interactions between the positively charged region of SDF-1α and negatively charged sulfate groups of heparin [54, 56]. Besides, the sustained release property of the loaded protein is also improved due to the improved bonding strength. Furthermore, the influence of the loading capacity on release profile should not be ignored. Generally speaking, the more protein contained within the scaffold the stronger the burst release will be. Some researchers have noted that the concentration of SDF-1α around heparin-mediated scaffolds influences cell migration [15, 16, 64]. Thus, it is necessary to ensure that the dose of loaded SDF-1α matches the regeneration process of the injured tissue.

In short, immobilization of SDF-1α through specific heparin-mediated interactions is widely used in scaffolds for *in situ* tissue engineering due to its strong interaction, efficient adsorption and reduced degradation. Nevertheless, the bonding process is relatively complicated.

### Particulate systems

Micro/nano particles carriers are widely employed in fabricating controlled drug delivery systems. The direct loading or adsorption of a chemokine into a particulate system is relatively convenient to achieve. However, it is associated with various issues such as high burst release, protein aggregation and denaturation. In order to prevent the burst release, some investigators employed microemulsion spheres to load the chemokine. In particular, Cross and colleagues [65] incorporated SDF-1α with poly(lactide-co-glycolide) (PLGA) microspheres using a double emulsion solvent extraction/evaporation technique to achieve sustained release of SDF-1α over 50 days. Additionally, Wu et al. [66] applied carboxyl-polyethylene glycol-4000-carboxyl (COOH-PEG4000-COOH) to the surface of their microemulsion spheres. This allowed SDF-1α to bind the microparticle through amide bonds, enhancing loading efficiency. The loaded chemokine could be released from the micro emulsion bubble by directed ultrasonic waves. However, utilizing emulsification techniques to fabricate protein-encapsulated particles may result in protein denaturation and the loss of bioactivity, due to exposure of protein to organic-aqueous interfaces [26]. Zamani et al. [27] introduced coaxial electrospraying to prevent protein denaturation during the fabrication process by reducing the contact time of the bioactive factors with other reactants.

In addition, micro- or nanoparticle-incorporated hydrogels have also been widely investigated to control the delivery of chemokines for tissue engineering applications, such as bone or cartilage regeneration [37, 67, 68]. Both temporally and spatially controlled release of these bioactive molecules in specific sites have been proved, thus being valuable in modulating the behavior of encapsulated cells. Nevertheless, the loss of bioactivity in encapsulated molecules due to high temperatures, organic solvents, and/or shear stress during the fabrication of the micro- or nanoparticles is likely unavoidable.

Recently, In order to create smart scaffolds that control chemokine release with time-specific, site-specific and rate programmed characteristics, some environmental stimuli-responsive microcapsules have been researched [69–71]. Chen et al. [20] developed a delivery system to control the release of SDF-1α by making microcapsules containing thermo-sensitive polymeric gates on their outer pore surfaces. The pore surfaces of the microcapsules was grafted by poly(N-isopropylacrylamide) (PNIPAAm) using plasma-graft pore-filling polymerization. The grafted PNIPAAm were in swollen state at ambient temperature, causing the pores in the outer surfaces to be blocked and thus the release rate of SDF-1α was low. While the temperature was above 22 °C, grafted PNIPAAm were in shrunken state, causing the pores in the outer surfaces to be opened, with a corresponding increase in the release rate of loaded SDF-1α. In addition to PNIPAAm, a copolymer called poly(polyethylene glycol citrate-co-N-isopropylacrylamide) (PPCN) is also characterized by its thermoresponsive behavior, antioxidant properties and morphology, and has received attention in protein delivery [72]. Kim et al. [73] has employed a pH sensitive copolymer named poly (urethane amino sulfamethazine) (PUASM) to load SDF-1α. The polymer forms micelles and encapsulates proteins effectively via ionic interaction at physiological pH. At environmental pH lower than 5.5, the micelle disassembles due to the ionization of tertiary amines, releasing the encapsulated protein.

Self-assembled monolayer deposition is often used to fabricate heparin-coated nanoparticles that could be utilized to load SDF-1α through specific heparin-mediated interactions. Specifically, a polyelectrolyte layer can be deposited onto an oppositely charged substrate through electrostatic adsorption. Na et al. [74] developed a heparin/poly(L-lysine) self-assembled nanoparticle-immobilized PLGA microsphere system, and showed that the specific binding activity of heparin allowed the loading of different bioactive factors. Wang [18] discovered that load capability and release kinetics of bioactive factors immobilized on self-assembled particles can be modified simply by changing the ratio of heparin to polymer.

At present, some investigators have proposed the use of particulate systems to load multiple bioactive factors by physical absorption to facilitate tissue repair in the body [75]. Richardson et al. [76] investigated a tissue-specific delivery system to deliver two or more bioactive factors. The first approach involved simply mixing lyophilized vascular endothelial growth factor (VEGF) with polymer particles before processing the polymer into a porous scaffold. The second approach involved pre-loading the bioactive factor in PLGA microspheres, and then fabricating scaffolds with these particles. These approaches provided distinct release kinetics for each bioactive factor. A composite scaffold comprising drug-loaded fiber and bioactive factor-loaded microspheres was prepared by simultaneous electrospinning and electrospraying in our recent work. The in vitro release test showed that the release properties of the drug and the bioactive factor were distinct (unpublished observation). In fact, the multiple bioactive factors-loaded particle system(including SDF-1α) has been investigated by many researchers (Table 2). However, the optimization of the synergistic factors to promote the tissue regeneration process remains to be done.

**Table 2:** Synergistic effect between SDF-1α and other bioactive factors

Bonding strategy	Factors	Implant position	Scaffold	References
Direct loading or adsorption (Adsorb solution through inject)	SDF-1α and BMP-2	Mouse, calvarial defects	Commercial collagen^a^	[94]
			Gelatin hydrogels	[95]
Direct loading or adsorption (Direct loading during manufacture process)		Mouse, ulna critical-sized defect	Degradable hydrogels	[96]
		Mouse, calvarial defects/ subcutaneous sites	Commercial collagen^b^	[97]
	SDF-1α and VEGF	Mouse, calvarial defects/ subcutaneous sites	Commercial collagen^b^	[97]
	SDF-1α and platelet-derived growth factor (PDGF)	Mouse, calvarial defects/ subcutaneous sites	Commercial collagen^b^	[97]
	SDF-1α and simvastatin	Mouse, calvarial defects	PLGA	[117]
	SDF-1α and insulin-like growth factor-1 (IGF-1)	Rat, lateral gastrocnemius muscle of the TK-injured limb	PEGylated fibrin gel matrix	[98]

^a^Resorbable atelocollagen sponges (Teruplug; Terudermis Olympus Terumo Biomaterials Co.)

^b^Collagen scaffold (Geistlich Pharma AG)

In summary, particulate systems are able to control the release kinetics of bioactive factors. Furthermore, the system allows multiple factors to be loaded into scaffolds efficiently.

## Applications

### Vascular scaffolds

Replacement of diseased arteries is a common treatment. More than 500,000 vascular grafts are used for coronary artery or peripheral arterial replacement each year [47]. However, autologous arterial and venous graft material has already been deployed, or is simply unusable may not be available in many cases [77]. Frequent occlusion and thrombosis in smaller grafts (<6 mm) limits the application of synthetic vascular grafts. Furthermore, the long-term patency rate of the synthetic vascular grafts is rather low due to the lack of endothelialization [47]. Tissue engineered vascular grafts are typically cell-based constructs. However, harvesting vascular cells, in vitro cell culture and making the grafts may take months. Therefore, *in situ* regeneration approach that recruits host cells is attractive.

SDF-1α is a promising chemoattractant of host EPCs and MSCs because it induces host progenitor cell mobilization and recruitment by binding to receptors CXCR4 and CXCR7. However, direct injection of SDF-1α is problematic. The short circulation half-life and extraneous interactions with multiple binding sites all reduce its local concentration.

Thus, it is vital to control the release of SDF-1α from vascular grafts by using an appropriate bonding method. Heparin could prevent thrombus formation [16, 47, 55], and also serve as an adapter for SDF-1α binding. For instance, Yu et al. [47] used NH_2_-PEG-NH_2_ to link heparin with polymer scaffolds, and then immobilized SDF-1α. Compared with physically-adsorbed SDF-1α, heparin-bond SDF-1α was more stable and demonstrated sustained release of SDF-1α. Furthermore, the in vivo test revealed that the inner surfaces of the graft were covered by endothelial cells that had differentiated from EPCs. Six months post implantation, many microvessels were found in the outer part of the scaffolds indicating that heparin + SDF-1α treated grafts had been well vascularized.

Lee et al. [51] used heparin and a polycation to form a coacervate that was incorporated into PGS scaffolds. This strategy also provided long-term sustained release of SDF-1α in open porous structured vascular scaffolds, which favored vascular regeneration. Finally, SDF-1α-containing nanoparticles have also been used for vascular grafts because of their sustained release characteristics [76, 78].

SDF-1α-loaded vascular grafts have many advantages, such as cell-free and available off-the-shelf. However, modulation of the release property of SDF-1α on grafts to match the rate of regeneration in vivo is still challenging.

### Articular cartilage scaffolds

Articular cartilage defects can be classified as partial-thickness, full-thickness and osteochondral defects. Partial-thickness defects are the defects in the surface of articular without penetratingthe tidemark, while osteochondral defects are those that penetrate through the tidemark and subchondral bone until the bone marrow. Full-thickness defects are between the tidemark and bone marrow. Researchers found that osteochondral and full-thickness defects can heal spontaneously [79, 80] while partial-thickness defects cannot [81–83], which is attributed to the migration of stromal cells from bone marrow. Thus, It can be inferred that recruiting stem cells especially bone marrow stem cells (BMSCs) after articular cartilage damages is important to rebuild the defects.

Wei et al. [84] believe that bone marrow secreted SDF-1α around the subchondral bone is the key point to affect the self-repair ability compared with full-thickness, osteochondral defects and partial-thickness defects. Zhang et al. [85] presented an effective strategy to create an *in situ* matrix environment by implanting an SDF-1α-containing type one collagen (Col1) scaffold. Col1 or Col1 + SDF-1α scaffold were employed to coverpartial-thickness defects created on the patellar groove of rabbits. Meanwhile, untreated defects were regarded as control group. The Col1+ SDF-1α group had a significantly higher histological macroscopic score for moderate neo-tissue coverage, surface regularity, and a smoother connection with the host cartilage. This revealed that the matrix environment created by SDF-1α loaded Col1 scaffold did improve the spontaneous regeneration capacity of partial-thickness defects.

Compared with the self-healing process of articular cartilage, the regenerated tissue treated by SDF-1α scaffold has mechanical properties that are more similar to the original. Sukegawa et al. [86] used SDF-1α-loaded alginate gel to repair osteochondral defects. A full-thickness osteochondral defect was created in the patella groove of the distal femur in rabbits. The compressive modulus of regenerated tissues and the histological scores demonstrated prominent improvement compared with the blank control group.

One of the current limitations of bone and cartilage tissue engineering is the lack of sufficient blood supply in the initial phase following implantation [87]. Meanwhile, vascularization of the implant proceeds slowly and only a few blood vessels reach the center of the scaffold after several weeks [88]. Inadequate vascularization following implantation results in nutrient deficiency, which then leads to cell death in the tissue-engineered scaffolds [89, 90]. In order to efficiently enhance migration of vascular cells into the scaffold, Chen et al. [91] fabricated a collagen scaffold with radially oriented channels and investigated its cell recruit property in combination with SDF-1α. They found that cells infiltrated further into the center of the scaffold. Besides, Brouwer and his colleagues also designed a scaffold with radial pore structure to repair the diaphragm defects, and reached the same conclusion [92, 93]. The in vivo experiments on rabbits confirmed that BMSCs could also be recruited into the radially-oriented scaffold with the assistance of SDF-1α.

Although SDF-1α loaded articular cartilage scaffolds have been widely researched, the regenerated tissue is still different from natural articular cartilage both in structural constitution and mechanical properties. Further study is necessary to optimize the structure and component of the scaffolds, as well as loading capacity and release property of SDF-1α.

### Osseous scaffolds

Currently, SDF-1α-loaded scaffolds have been widely used to repair bone defects (Table 2), and there is an increasing amount of work addressing the synergy of SDF-1α with other bioactive factors for bone repair [15, 94–98].

Ratanavaraporn et al. [28] evaluated the activity of gelatin hydrogels combined with SDF-1α and bone morphogenetic protein 2(BMP-2) on bone regeneration at an ulna critical-sized defect of rats. The result demonstrated that a SDF-1α and BMP-2-loaded scaffold was more effective to induce bone regeneration than a scaffold loaded with either factor alone. Other researchers also found the same effect and provided some possible explantion. On the one hand, synergetic effect of SDF-1α and BMP-2 may influence the SDF-1α/CXCR4 or other signal pathways to enhance cell recruitment around scaffold. On the other hand, the enhanced recruitment of HSCs improves the vascularization, which helps to supply nutrient [99–101]. Furthermore, the SDF-1α and BMP-2 signaling may activate osteogenic differentiation, which improve the bone regeneration [102, 103].

It is well known that several members of the BMP family, including BMP-2, −4,−6, −7, and −9, can induce MSCs to undergo osteogenic differentiation and promote bone formation [104–107]. However, using BMPs has some disadvantages, including ease of degradation and high cost [108–110]. Simvastatin (SIM) is a competitive inhibitor of 3-hydroxy-3-methyl coenzyme A (HMGCoA) reductase, which improves the osteogenesis of adipose-derived stromal cells (ASCs) [111]. Meanwhile there have been many studies demonstrating the promotion of bone regeneration by the local application of SIM with different delivery systems in various animal models [112–115]. Furthermore, SIM has recently been shown to mobilize MSCs migrating to bone defects or areas of spinal cord injury [116]. Thus, Liu et al. [117] fabricated a PLGA-based cell-free bone tissue engineering scaffold loaded with SIM and SDF-1α, and applied it in critical-sized calvarial defects in mice. Their findings suggest that the combination of SDF-1α and SIM increases MSCs migration and homing, promotes angiogenesis and enhance the expression of BMP-2 in newly-formed bone tissue.

Richardson et al. [76] investigated a polymeric system that allowed for the tissue-specific delivery of two or more bioactive factors with controlled dose and release rate. Briefly, a porous PLGA scaffold loaded with multiple growth factors was fabricated by a high-pressure carbon dioxide fabrication process. Two types of strategy were used to load bioactive factors, processing the polymer into a porous scaffold. One bioactive factor was simply mixed with polymer particles which lead to rapid release. Another bioactive factor was pre-encapsulated in PLGA microspheres which lead to a comparatively slower release rate. The scaffold was fabricated with these particles.

The therapeutic effect of multiple bioactive factors contained within the scaffold system was more dramatic than the single factor system. Nevertheless, there are still many problems which limit its development. For instance, the preparation process of the scaffold with its multiple bioactive factors is complicated, and its molecular mechanism and the safety of the system remain to be investigated. In order to mimic the process of natural bone healing, the ideal smart multiple bioactive factors loaded scaffold system should control the release sequence as well as the release rate of each factor.

### Myocardium scaffold and other applications

The deterioration of cardiac function following myocardial infarction (MI) is a major cause for high mortality due to heart disease [118]. It is important to ensure myocardium regenerates after MI. As for cell therapy, poor cell engraftment in the myocardium limits the efficiency of using stem/progenitor cells to treat MI [31]. Investigators have demonstrated that myocardial SDF-1α expression is temporarily increased following MI [119, 120]; however, long-term SDF-1α release is necessary for cardiac regeneration.

As for SDF-1α-loaded MI scaffolds, achieving sustained release and preventing premature enzymatic degradation of the loaded chemokine are critical. Zhang et al. [32] introduced a PEGylated fibrin patch to deliver a sustained flux of SDF-1α to an acute MI (AMI) site. Specifically, PEGylated fibrin patch was fabricated by mixing thrombin with SDF-1α incorporated PEGylated fibrinogen. An in vitro study demonstrated that SDF-1α was successfully released from the patch over 10 days. The in vivo release study in mouse MI model indicated that the controlled release of SDF-1α from a PEGylated fibrin patch significantly recruited more c-kit + cells to the infarct area at the second week than did the direct injection group. This phenomenon was observed for up to 4 weeks following implantation. It revealed that SDF-1α contained within a PEGylated fibrin patch could overcome premature degradation and it healed AMI through sustained chemokine release. Some researchers have adopted SDF-1α-linked hydrogel to achieve a long release duration and high loading efficiency for MI treatment [16, 31, 121].

Despite recent research showing SDF-1α-loaded MI scaffolds could help to repair heart injury following MI, it remains a challenge to determine the best release property, especially the concentration of the chemokine around the scaffold for safe and efficient treatment.

This review only covers applications in several tissues. There are also studies on other tissue injures such as skin ulcers [49, 122], traumatic brain injury [123], and intervertebral disc degeneration [33] because of the ability of SDF-1α-loaded scaffolds to recruit cells.

## Conclusions

SDF-1α-loaded scaffolds have been adopted to investigate the regeneration of blood vessels, myocardium, cartilage, bone and many other tissues*.* Most investigators adopt physical immobilization to load SDF-1α, especially direct loading or adsorption due to its ease of operation. Furthermore, immobilization of cues through the formation of ionic complexes is appropriate for the loading of SDF-1α for its universality, relative strong interaction, lack of linker molecules, reduced dependence on surface properties, and the protection of immobilized factors from inactivation. However, the disadvantages of physical immobilization are also clear. Uncontrolled burst release and short release duration limit its application. In order to restrain the burst release and prolong the release duration of SDF-1α from scaffolds, some researchers have introduced heparin-mediated immobilization. For example, an amidation reaction between heparin and scaffold has been utilized. SDF-1α is then incorporated into the scaffold through the specific heparin-mediated interaction. Furthermore, micro-carrier immobilization can also provide sustained release of SDF-1α by loading bioactive factors into nano- or micro-polymer particles. The SDF-1α-loaded particle is mixed with raw materials to fabricate the scaffolds or simply coated on the surface of scaffolds. The most remarkable merit of the particulate system is that it provides microcarriers to load multiple bioactive factors that may promote effective cell migration, growth and differentiation. These bonding strategies may also be expanded to immobilize other chemokines or growth factors. For in vivo applications, it is critical to prevent the enzymatic degradation of SDF-1α upon release from the scaffolds. Therefore, protease-resistant SDF-1α may have potential applications for *in situ* tissue regeneration.

It has been found that the release kinetics, loading efficiency and cell homing capability of SDF-1α-loaded scaffolds depend on their bonding strategies. To mimic a physiological cellular microenvironment, one needs to consider the nature of the bonding strategy the scaffold should adopt for its application. It is likely that a SDF-1α-loaded scaffold may be loaded with multiple bioactive factors through a combined use of different bonding strategies, in which synergistic effects of the bioactive factors can arise. Specifically, it may become a future trend to control the release sequence as well as the release rates of the multiple bioactive factors by choosing appropriate bonding strategies for each bioactive factor.

## Abbreviations

ASCs: Adipose-derived stromal cells
BMP-2: Bone morphogenetic protein 2
Col1: Type one collagen
CXCR4: CXC chemokine receptor type 4
DPP-4: Dipeptidylpeptidase-4
EPCs: Endothelial progenitor cells
HMGCoA: 3-hydroxy-3-methyl coenzyme A
HSCs: Hematopoietic stem cells
IEP: Isoelectric point
MI: Myocardial infarction
MMPs: Metalloproteinases
MSCs: Mesenchymal stem cells
PGS: Poly(glycerol sebacate)
PLGA: Poly(lactide-co-glycolide)
PNIPAAm: Poly(N-isopropylacrylamide)
PPCN: Poly(polyethylene glycol citrate-co-N-isopropylacrylamide)
PUASM: Poly(urethane amino sulfamethazine)
SDF-1α: Stromal cell-derived factor 1α
SIM: Simvastatin
sPEG: Star-poly(ethylene glycol)
VEGF: Vascular endothelial growth factor
